# Effect of Androgen Deprivation Therapy on the Results of PET/CT with 18F-Fluciclovine in Patients with Metastatic Prostate Cancer

**DOI:** 10.3390/tomography8030120

**Published:** 2022-06-03

**Authors:** Tore Bach-Gansmo, Katrine Korsan, Trond Velde Bogsrud

**Affiliations:** 1 PET Imaging Centre, University Hospital of North Norway, 9019 Tromsø, Norway; trond.bogsrud@unn.no; 2 Department of Diagnostic Imaging and Intervention, AHUS University Hospital, 1478 Lørenskog, Norway; 3 Department of Research Support for Clinical Trials, Clinical Trials Unit (CTU), Oslo University Hospital, 0424 Oslo, Norway; katrko@ous-hf.no; 4 Department of Nuclear Medicine and PET-Centre, Aarhus University Hospital, 8200 Aarhus, Denmark

**Keywords:** PET, prostate cancer, androgen deprivation therapy, fluciclovine

## Abstract

Background: 18F-fluciclovine is a positron emission tomography (PET) radiotracer approved for the detection of prostate cancer recurrence. No effect of androgen deprivation therapy (ADT) on its performance has been established. Purpose: To study the impact of concurrent ADT on disease detection with 18F-fluciclovine PET in patients with prostate cancer. Materials and Methods: Data from patients with prostate cancer who had been receiving ADT for ≥3 months at the time of undergoing an 18F-fluciclovine PET/CT at our institution were retrospectively reviewed. Seventy-three scans from 71 patients were included. The scans indicated rising prostate-specific antigen (*n* = 58), staging advanced disease (*n* = 4) or therapeutic monitoring (*n* = 9). Patients’ medical records provided baseline clinical data and post-scan outcomes (median follow-up 40 months). Results: Malignant lesions with increased uptake of 18F-fluciclovine were detected in 60/73 (82%) scans; 33 (45%) had lesions in the prostate/bed and 46 (63%) in extraprostatic sites. Patients received ADT for a median of 2 years (range 3 months to >10 years) pre-scan. The time on ADT did not influence detection; the detection rates were 89% for patients who had received ADT for <1 year, 63% for a treatment period of 1–<2 years, 83% for 2–4 years, 78% for >4–10 years, and 67% for a treatment period of >10 years. Conclusion: 18F-fluciclovine detected recurrent or metastatic lesions in 82% of patients with prostate cancer receiving ADT. The rates achieved in the present study are consistent with widely reported data for 18F-fluciclovine PET/CT, suggesting that withdrawal of ADT before scanning is not necessary.

## 1. Introduction

Anti-1-amino-3-[18F]-fluorocyclobutane-1-carboxcylic acid (18F-fluciclovine; Axumin^®^, Blue Earth Diagnostics Ltd., Oxford, UK) is an artificial amino acid-based positron emission tomography (PET) radiotracer approved by the U.S. Food and Drug Administration and the European Medicinal Agency for the detection of local recurrence and metastases in patients with prostate cancer and rising blood level of prostate-specific antigen (PSA) after initial radical treatment [1]. 18F-fluciclovine is an analogue of the amino acid L-leucine, cyclized into a cyclobutane ring and labeled as 18F. Cell uptake of 18F-fluciclovine is dependent on sodium-dependent amino acid transporters (ASCT) such as ASCT1 and ASCT2, and, to a lesser extent, sodium-independent LAT1, LAT2 and SNAT2 transporters [2,3,4,5,6]. Despite utilizing amino acid transporter systems for uptake, once inside the cell, 18F-fluciclovine is neither metabolized nor used for protein synthesis. 18F-fluciclovine follows the concentration gradient to reach the maximum intracellular concentration within a few minutes after injection, and is then excreted following the inverse concentration gradient [2,3,4,5,6].

Androgen deprivation therapy (ADT), which is a standard treatment for metastatic prostate cancer, is known to impact multiple amino acid transporters. In relation to 18F-fluciclovine uptake, ADT is known to cause secondary upregulation of ASCT1 and LAT1 [7]. ADT is known to have an inhibitory effect on the uptake of radionuclide-labeled choline in patients with androgen-sensitive prostate cancer [8,9,10], but no impact on the uptake of 18F-fluciclovine has been established, particularly in patients with castration-resistant prostate cancer. 

A substantial proportion of false-negative 11C- or 18F-labeled choline PET/CT has been reported in patients receiving ADT [8,9,10]. Fuccio et al. reported that 11C-choline PET scans that were initially positive for metastatic spread in 13 out of 14 patients became negative in 9 patients following 6 months’ treatment with ADT [8].

In light of the data reported for radionuclide-labeled choline PET/CT, the present study explored the impact of ADT on the detection rate of 18F-fluciclovine PET/CT to help elucidate whether or not it is necessary to withdraw ADT from patients with prostate cancer before PET/CT scanning with 18F-fluciclovine.

## 2. Materials and Methods

Data from 457 patients who underwent 18F-fluciclovine PET/CT for prostate cancer at our institution between 2014 and 2018 were retrospectively reviewed. Only patients whose referring physician reported that the patient was currently receiving ADT and had been for at least 3 months prior to imaging were included. Patients’ medical records were reviewed in order to confirm the use of ADT. The pre-scan plasma PSA level and Gleason score at the time of the primary diagnosis were also recorded for each patient. In order to explore any impact of 18F-fluciclovine PET/CT, patient records were reviewed for up to 45 months (median 40 months, range 18–45 months) after the 18F-fluciclovine PET/CT. 

In line with local regulations, the study protocol was reviewed by the local ethics committee and approved by the hospital’s data protection officer. Written informed consent was obtained from all patients and included a statement that indicated permission for the use of data for research purposes. 

In total, data from 71 patients (median age, 69 years; range, 47–85 years), contributing to 73 scans, were included. The patient’s baseline characteristics are summarized in Table 1 and the Gleason scores at the time of primary diagnosis are presented in Table 2. 18F-fluciclovine PET/CT was conducted a median of 7 years after radical prostatectomy (*n* = 29) and a median of 6 years after radiotherapy (*n* = 30). The majority of 18F-fluciclovine PET/CT scans (*n* = 58) indicated rising PSA, despite ADT. Restaging (*n* = 4) and therapeutic control (*n* = 9) were less common indications. The underlying indication for 18F-fluciclovine PET/CT was not routinely recorded in patient referral, but when planning for potential salvage surgery, salvage radiotherapy or bone lesion-targeted radiotherapy were common. 

### 2.1. 18F-Fluciclovine PET/CT

18F-fluciclovine was manufactured by automated radiosynthesis and administered at approximately 370 MBq. Imaging was captured by a Siemens Biograph mCT40 (Siemens Healthineers, Erlangen, Germany) using time-of-flight, point spread functions for resolution recovery, and iterative reconstruction (2 iterations, 21 subsets). Low-dose CT without contrast enhancement was used for attenuation correction and anatomical correlation. For all but four patients, imaging was started immediately post-injection with a 5 min dynamic list-mode acquisition over the pelvis. The dynamic acquisition of a 5 min 1-bed position over the pelvis was followed by a further 4–5 bed, 2 min acquisition as detailed in Table 3. 

### 2.2. 18F-Fluciclovine PET/CT Interpretation

For all patients, a dedicated pelvic MR and/or a diagnostic CT with intravenous contrast from thorax through pelvis was available for correlation with the findings on PET/CT. 18F-fluciclovine PET/CT images were evaluated by experienced PET/CT readers who interpreted images according to 2019 EANM/SNMMI procedure guidelines [11]. Briefly, specific anatomic locations were classified as either “positive” or “negative” for malignancy on the basis of visual assessment of non-physiological activity, with lesions suspicious for metastases marked as “positive”. Any lesions considered indeterminate after comparison with CT and/or MR were scored as “negative”. Scans that had no suspicious lymph nodes, bone lesions, prostate lesions or lesions in the prostate bed were reported as “negative”. Imaging positivity rate (detection rate) was defined as the proportion of scans containing one or more lesions considered “positive” for prostate cancer. Detection rates were determined for the prostate bed and for extraprostatic sites (regional lymph nodes and distant metastases).

## 3. Results

**Figure 1:**
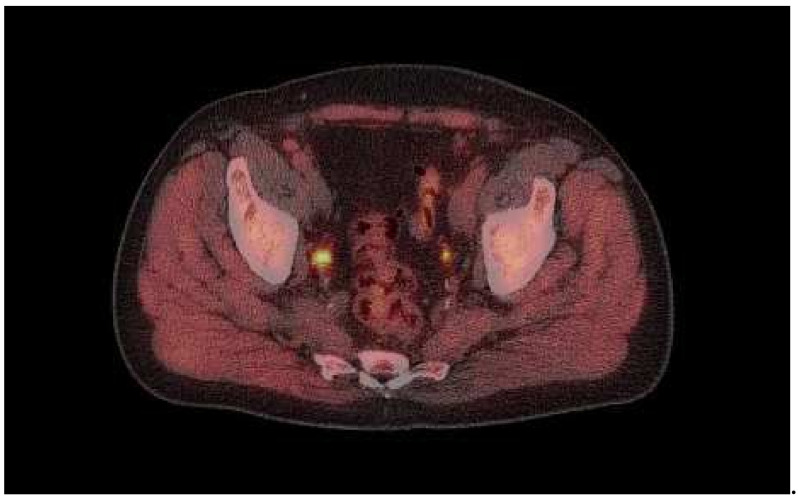
18F-fluciclovine PET example image.

Of the 73 scans, 60 (82%) showed 18F-fluciclovine-positive lesions. In total, 33/73 (45%) of the scans showed localized uptake consistent with malignancy (“positive” findings) in the prostate or prostate bed, and 46/73 (63%) showed lesions outside of the prostate or prostate bed (example case provided in Figure 1). Extraprostatic disease included lymphatic spread in 38/73 (52%) and bone lesions in 5/73 (6.8%). No pulmonary or liver metastases were detected. Sclerotic bone lesions without increased tracer uptake were observed in two patients. One of these had no other 18F-fluiciclovine findings, while the other patient had increased uptake in pelvic lymph nodes, which was suspicious for malignancy.

Image captured from a 74-year-old male showing ^18^F-fluciclovine-avid lesion in right side iliaca interna lymph node (SUV 12).

The patient had undergone robotic-assisted laparoscopic prostatectomy in 2010 and salvage surgery in 2014, following a PSA rise to 12 ng/mL. Post-salvage surgery, his PSA was 0.3 ng/mL, but this slowly increased until bicalutamide was prescribed, initially intermittently, but continuously for the 9 months prior to the scan. PSA at the time of scanning was 4.9 ng/mL.

Further evaluation of the “negative” PET/CT scans (*n* = 13) revealed that 5/13 (38%) were from patients with a PSA level below 0.5 ng/mL, two were from patients with missing PSA data, and one was from the patient described above with sclerotic bone lesions without increased tracer uptake, which may have represented a healed metastasis.

At the time of the 18F-fluciclovine PET/CT, the cohort had been receiving ADT for a median of two years (range, 3 months to >10 years). A broad range of ADT was prescribed; the most commonly prescribed ADT was bicalutamide (Casodex^®^, AstraZeneca, Cambridge, UK; *n* = 30). Eighteen patients were prescribed goserelin (Zoladex^®^ AstraZeneca), seven received goserelin plus bicalutamide, while the remainder received degarelix (Firmagon^®^ Ferring Pharmaceuticals, St-Prex, Switzerland), enzalutamide (Xtandi^®^ Astellas Pharmaceuticals, Tokyo, Japan), leuprorelin (Eligard^®^, Astellas and Procren^®^, AbbVie Logistics, Zwolle, Holland) and abiraterone (Zytiga^®^, Janssen Pharmaceuticals, Beerse, Belgium), either alone, but more commonly in various combinations. As shown in Table 4, the 18F-fluciclovine detection rate was not influenced by the length of time on ADT, and was consistently above 60% irrespective of the time on ADT.

At the end of the follow-up period, eight of the patients had died, but the cause of death could not be verified. Each of these eight patients had shown lesions with high 18F-fluciclovine uptake, with four patients having bone metastases.

The evaluation of data from the nine patients who had been referred for 18F-fluciclovine PET/CT, in order to monitor their response to ADT, showed that five had a good response with a “negative” 18F-fluciclovine scan. ADT was subsequently dropped for only one of these patients. He remained free of signs of relapse at his last recorded monitoring (18 months after ADT was stopped). Four of these patients remained on ADT, and three showed a persistent response 2–3 years later with PSA values <0.1 ng/mL for two and 0.4 ng/mL for the third patient. One patient showed a slow continuous rise in PSA and a follow-up 18F-fluciclovine PET/CT 16 months later showed high uptake of 18F-fluciclovine in mediastinal lymph nodes, consistent with metastases.

## 4. Discussion

We have performed a retrospective review of 18F-fluciclovine PET/CT data from patients with metastatic prostate cancer undergoing treatment with ADT. We found detection rates in this patient cohort treated with a large variety of ADT to be in line with our data published earlier from this patient population [1]. 

It is expected that patients who have been prescribed ADT have more extensive and aggressive disease than those not prescribed ADT [12]. Identifying the sites of recurrence and determining the extent of disease will be important in this patient group as salvage surgery, salvage radiation therapy or localized treatment may still be required to optimize the patient’s management.

As ADT is known to impact some of the transporters involved in the uptake of 18F-fluciclovine into prostate cancer cells [7], the uptake of 18F-fluciclovine PET could be impaired in patients receiving ADT, as is the case for ADT inhibition of the uptake of 11C- and 18F-choline [8,9,10]. 

The present study did not aim to draw comparisons between the utility of 18F-fluciclovine in patients receiving and not receiving ADT, but rather compare the findings with previously published data [1]. The present data show that 18F-fluciclovine PET/CT remains a useful tool in patients receiving ADT, with an overall lesion detection rate of 82%. Previous studies have established the effectiveness of 18F-fluciclovine in the detection of recurrent prostate cancer [1,13,14]. However, whether or not the patients in those studies were receiving ADT at the time of scanning was not recorded consistently. The present study comprised a cohort of patients who had all received ADT for more than 3 months, with some having received treatment for more than 10 years. We show that the length of time on ADT does not affect the rate of detection. Moreover, our overall patient-level 18F-fluciclovine detection rate of 82% (95% CI, 72–91%) compares favorably with the 18F-fluciclovine detection rate (68% [95% CI, 64–72%]) from a multisite retrospective study that included data from our institution [1], and with several other studies that reported the overall patient-level detection rate to be in the range of 56–83% [13,15,16]. This goes some way to suggest that concomitant ADT does not substantially impact the performance of 18F-fluciclovine PET/CT in patients with prostate cancer.

Recently, it has been suggested that PET tracers that target prostate-specific membrane antigen (PSMA) may be affected by ADT-induced early, but temporary, upregulation of PSMA expression, which then becomes downregulated with prolonged ADT use [17]. No similar effect appears to impact 18F-fluciclovine imaging, and so it may offer a reliable imaging option for patients with prostate cancer who are undergoing management with ADT. The European Association of Urology guidelines suggest that with PSMA imaging with biochemical recurrence at PSA levels above 0.2 ng/mL, there is less or no need to upstart ADT therapy before an eventual PSMA PET [18].

The findings of the present study are of clinical importance. In contrast with 11C- or 18F-choline PET, the present data offer evidence suggesting that ADT has no substantial impact on the detection rate of prostate cancer recurrence with 18F-fluciclovine PET. Currently, there is no conclusive answer to whether or not ADT should be withdrawn before 11C- or 18F-choline PET. Ceci et al. [19] showed that, when performed during ADT interruption, 11C-choline PET/CT was able to detect sites of recurrence in patients for whom a prior scan was conducted when ADT was “negative”. The present study, however, indicates that when PET imaging is conducted with 18F-fluciclovine, there might be no need to withdraw ADT from the patient beforehand. Indeed, such withdrawal would, in many cases, be undesirable from a clinical viewpoint.

The most important limitation of this retrospective study was that we were not able to compare 18F-fluciclovine PET studies in patients on ADT and after withdrawal. It is, thus, uncertain if an interruption of ADT would have allowed an even higher detection rate. Nevertheless, our data provide substantial evidence to warrant future prospective trials. A further limitation was that we were unable to conduct histopathologic confirmation of positive lesions, largely because this was a retrospective study and histopathologic confirmation is rarely ordered for patients receiving ADT at our institution. In addition, in patients with low PSA levels, PET-positive lesions can be difficult to biopsy, owing to their small size or the presence of a lymph node that is not easily accessible by surgery.

## 5. Conclusions

In conclusion, our observation was that the 18F-fluciclovine PET/CT detection rates in patients with recurrent prostate cancer receiving ADT at the time of the scan are similar to or higher than the rates previously reported for 18F-fluciclovine PET/CT. This suggests that it might not be necessary to withdraw ADT from patients with prostate cancer before 18F-fluciclovine PET scanning. Indeed, it is reasonable “good clinical practice” not to discontinue ADT in this particular setting since the detection rate is already very high.

## Figures and Tables

**Table 1:** Patient characteristics (n = 73) at the time of referral for 18F-fluciclovine PET/CT.

Patient Characteristics	Number of Patients (%)
Primary therapy	
Radical prostatectomy External beam radiation therapy - Alone - With cryotherapy Primary hormonal treatment Missing data	29 (40%) 30 (41%) 28 (38%) 2 (2.7%) 9 (12%) 5 (7%)
**Last recorded PSA (ng/mL) before PET/CT**	
<0.5	5 (6.8%)
0.5–2	17 (23%)
>2–6	12 (16%)
>6–20	15 (21%)
>20	19 (26%)
Missing data	5 (7%)

PET/CT, positron emission tomography/computerized tomography; PSA, prostate-specific antigen.

**Table 2:** The Gleason scores of the cohort at the time of primary diagnosis.

Gleason Score	n (%)
*5–6*	2 (2.7%)
*7*	26 (36%)
*8*	16 (22%)
*9*	19 (26%)
*10*	2 (2.7%)
Missing data	8 (11%)

**Table 3:** PET/CT acquisition protocol.

Scanner	Siemens Biograph mCT40
Acquisition mode	3D
CT for attenuation correction and anatomic correlation	50 mA, 120 kVp
Target administered activity	370 MBq
CT contrast	No
Position	Supine
Direction	Pelvis to head
Arm position	Above head
Scan start position	Just below inguinal regions
Scan end position	Vertex
Minutes per bed position	See text
Number of bed position	6–8

CT, computerized tomography.

**Table 4:** Detection rate stratified by time on ADT.

Time on ADT	Number of Patients (n)	Positive Scan/n (%)
>3 months–<1 year	9	8/9 (89%)
1–<2 years	8	5/8 (63%)
2–4 years	24	20/24 (83%)
>4–10 years	23	18/23 (78%)
>10 years	6	4/6 (67%)
Unclear from records, but >3 months	3	1/3 (33%)

ADT, androgen deprivation therapy.

## Data Availability

The data presented in this study are available in an anonymous form on request from the corresponding author. The data are not publicly available being patient sensitive information.
